# Suppress the cell growth of cancer stem-like cells (NTERA-2) using Sox2-Oct4 decoy oligodeoxynucleotide−encapsulated niosomes-zinc hybrid nanocarriers under X-irradiation

**DOI:** 10.1016/j.heliyon.2024.e34096

**Published:** 2024-07-04

**Authors:** Behrooz Johari, Shabnam Tavangar-Roosta, Mahmoud Gharbavi, Ali Sharafi, Saeed Kaboli, Hamed Rezaeejam

**Affiliations:** a Zanjan Pharmaceutical Biotechnology Research Center, Zanjan University of Medical Sciences, Zanjan, Iran; b Department of Medical Biotechnology, School of Medicine, Zanjan University of Medical Sciences, Zanjan, Iran; c Nanotechnology Research Center, Medical Basic Scinces Research Institute, Ahvaz Jundishapur University of Medical Sciences, Ahvaz, Iran; d Pain Research Center, Ahvaz Jundishapur University of Medical Sciences, Ahvaz, Iran; e Department of Radiology Technology, School of Allied Medical Sciences, Zanjan University of Medical Sciences, Zanjan, Iran

**Keywords:** Sox2-Oct4, Cancer stem cells, Decoy ODNs, Radiation therapy, Niosomes, Zinc nanoparticle

## Abstract

Sox2 and Oct4 dysregulations could significantly increase in the cancer stem cell (CSC) population in some cancer cells and resistance to common treatments. In this study, the synergistic effects of Sox2-Oct4 decoy oligodeoxynucleotides-encapsulated Niosomes-zinc hybrid nanocarriers along with X‐irradiation conditions as a combinational therapy tool were investigated in the treatment of cancer-like stem cells (NTERA-2). The NTERA-2 cell line known as a cancer-like stem cell line was used in this investigation. Sox2-Oct4 decoy oligodeoxynucleotides were designed based on the sequence of the Sox2 promoter and synthesized. Physicochemical characteristics of ODNs-encapsulated niosomes-zinc hybrid nanocarriers (NISM@BSA-DEC-Zn) investigated with FT-IR, DLS, FESEM, and ODNs release kinetic estimation assays. Further investigations such as hemolysis, uptake, cell viability, apoptosis, cell cycle, and scratch repair tests were performed. All the above assays were completed with and without X-ray exposure conditions (fractionated 2Gy). Physicochemical characteristics results showed that the Niosomes-Zn nanocarriers were successfully synthesized. NISM@BSA-DEC-Zn was efficiently taken up by NTERA-2 cells and significantly inhibited cell growth, increased apoptosis, and reduced cell migration in both conditions (with and without X-ray exposure). Furthermore, NISM@BSA-DEC-Zn treatment resulted in G1 and G2/M cell cycle arrest without and with X-irradiation, respectively. The prepared nanocarrier system can be a promising tool for drug delivery in cancer treatment. Decoy ODN strategy along with zinc nanoparticles could increase the sensitivity of cancer cells toward irradiation, which has the potential for combinational cancer therapies.

## Highlights

• NISM@BSA-DEC-Zn nanocarrier have good hemocompatibility.
• *Cy3-labeled ODNs -loaded* nanocarrier *accumulate in NTERA-2 cells*.
• ODNs-loaded nanocarriers increase cytotoxicity and apoptosis rate in with and without X-irradiation exposure condition.
• ODNs-loaded nanocarriers induce cell cycle arrest at G0/G1 phase in no X-irradiation condition.
• ODNs-loaded nanocarriers induce cell cycle arrest at G2/M phase in X-irradiation exposure condition.

## Graphical abstract

**Figure:**
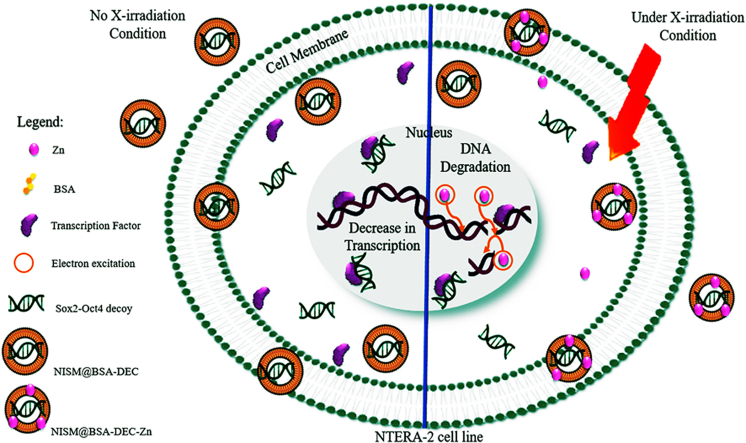

## 1 Introduction

Cancer continues to be a leading cause of death globally, necessitating the development of innovative strategies for treating its complex challenges [1]. Tumors consist of a diverse population of cells, including a subgroup called cancer stem cells (CSCs), which have significant roles in tumor initiation, progression, resistance to therapy, and recurrence [2,3].

Notably, the NTERA-2 cancer stem-like cell line, which exhibits pluripotent characteristics similar to CSCs [4], serves as a relevant model for studying mechanisms related to CSCs and potential therapies [5]. CSC activities are controlled by many intracellular and extracellular factors; these factors can be used as drug targets for the treatment of cancer [6].

Transcription factors Oct4, Sox2, and Nanog are implicated in maintaining pluripotency and are also involved in the development of tumors [7]. Oct4 (POU5F1) is one of the first transcription factors expressed in the fetus. Sox-2 is a member of the SRY family of HMG-box transcription factors and is expressed in many tumors. Both Oct4 and Sox2 are essential for self-renewal of stem cells, normal or cancer cells [8,9].

Conventional therapies such as surgery, chemotherapy, and radiation therapy have been widely used in many cancer treatments. Despite being widely used in clinics, radiation therapy has limitations, including treatment resistance and harmful effects on healthy tissues [10]. Along with conventional remedies, targeted therapy strategies including transcription factor decoy oligodeoxynucleotides (TFD) demonstrated promising opportunities for cancer treatment by inhibiting transcription factors in cancer cells [11,12]. Moreover, targeted inhibition of Sox2 and Oct4 transcription factors using decoy ODNs modulates the stemness properties of mouse embryonic stem cells [13].

In recent years, niosomes are a type of novel drug delivery system that can be used to carry both amphiphilic and lipophilic drugs. They are non-ionic surfactant-based vesicles that offer several advantages over liposomes, including stability, low toxicity, and lower cost. However, current approaches have limitations in maximizing the efficiency of drug delivery and targeting specific cellular pathways [14,15]. Niosomes have been extensively studied as a drug carrier for various medications, including anticancer and anti-infective agents. Different synthesis approaches have been explored to enhance the drug delivery capacity of niosomes, such as proniosomes, discomes, and aspasomes. Moreover, niosomes have been shown to have potential in targeted ocular, topical, and parenteral administration. The use of 3D printing technology allows for precise control over the size, shape, and composition of niosomes, which can enhance their drug-delivery capabilities [16,17]. In our previous study, the obtained results confirm that niosomes nanocarriers containing Nanog decoy ODNs can potentially suppress the metastatic ability of glioblastoma cells [18].

Moreover, although zinc nanoparticles (ZnNPs) demonstrate encouraging properties as radiosensitizers and cytotoxic agents against cancer cells, there are still challenges in effectively incorporating them into drug delivery systems and directing them toward CSCs [19]. In this particular context, we propose an innovative methodology to specifically target NTERA-2 cancer stem-like cells by utilizing a hybrid nanocarrier system consisting of niosomes and zinc nanoparticles that encapsulate decoy oligodeoxynucleotides (ODNs) that target Sox2 and Oct4 transcription factors [20].

The objective of this approach is to overcome the limitations of conventional therapies and existing drug delivery systems by simultaneously targeting CSC-related pathways and enhancing therapeutic effectiveness while minimizing unwanted effects on non-targeted cells. By combining the advantages of niosomes and ZnNPs, our approach capitalizes on their respective strengths while mitigating their weaknesses, thus presenting a promising strategy for CSC-targeted cancer therapy. Additionally, through the evaluation of the antitumor effects of this hybrid nanocarrier system under X-ray exposure conditions, we aim to gain insight into its potential as a combination therapy approach, which could provide new perspectives on overcoming treatment resistance and improving patient outcomes in cancer therapy.

## 2 Materials and methods

### 2.1 Reagents and materials

Reagents used for the synthesis of nanocarrier systems: BSA (CAS.9048-46-8), sorbitan monooleate or span80 (CAS.1338-43-8), polyoxyethylene sorbitan monooleate or tween 80 (CAS.9005-65-6), cholesterol (CAS.57-88-5), chloroform (Sigma Aldrich Co.), methanol (Sigma Aldrich Co.), and acetone (Emertat Co., Iran), Zn SO_4_ (Sigma-Aldrich Co., USA), ODNs (Bioneer Inc. Daejeon, Korea). l-Glutamine (Merck; CAS 56-85-9), Dulbecco's modified eagle's medium (DMEM) (D5796; Sigma), fetal bovine serum (FBS) (ES-020-B), MTT (57360-69-7), penicillin−streptomycin (P4333), trypsin-EDTA (Sigma-Aldrich), phosphate-buffered saline (PBS) (was prepared in the laboratory), Annexin V-FITC/PI kit (Sigma, USA), PI (Sigma−Aldrich), dimethyl sulfoxide (DMSO), cell culture plates (SPL Life Sciences).

### 2.2 Methods

#### 2.2.1 Cell culture

The NTERA-2 cell line (IBRC C10509) was purchased from the Iranian Biological Resource Center. NTERA-2 cancer cell line was cultured in DMEM medium containing 4.5 g/L glucose, 2 mM Glutamine, 10 % Fetal Bovine Serum (FBS), 100 units/mL penicillin, and 100 μg/mL streptomycin at 37 °C in a humidified atmosphere with 5 % CO_2_ incubator.

#### 2.2.2 Sox2-Oct4 decoy and scrambled ODNs design

Sox2-Oct4 decoy ODNs synthesized according to the promoter region of the related *Homo sapience* Sox2 gene [11]. Sox2-Oct4 decoy (DEC) and scrambled (SCR) ODNs sequences for synthesis ordered to Bioneer Inc (Korea). Also, sequences of SCR were designed by making mutations in the core binding site of Sox2-Oct4 ODN. Phosphorothioate (PS) modifications at 3′ and 5′ of ODNs sequence can protect ODNs from nucleases. Cy3 fluorescent dye was added at the 3′ terminus of ODNs to investigate the cell uptake efficiency of nanocarrier-containing ODNs into cells. In the sequence of designed ODNs, the core binding site, PS modifications, and mutations are shown by boldface, an asterisk (*), and underlining/italics, respectively.

Decoy ODNs sequences:

Forward: [5′‐G*CC**ATTGT**AATGCAATGT**ATTGT**GAT*G‐3′]

Reverse: [3′‐C*GGTAACATTACGTTACATAACACTA*C‐5′]

SCR ODNs sequences:

Forward: [5′‐G*CCA*GGC*TAATGCAATGTA*GGC*TGAT*G‐3′]

Reverse: [3′‐C*GGTCCGATTACGTTACATCCGACTA*C‐5′]

#### 2.2.3 Biosynthesis of ZnNPs

To synthesize ZnNPs, 4 % w/v of BSA was dissolved in ultrapure water under vigorous stirring. Then, 0.002 % w/v of ZnSO_4_ was added to the solution. After 20 min, 300 μL of NaOH 1 N was gently added drop by drop, and the temperature suddenly increased to 90 °C. The solution was left to react for 2 h, during which the color of the solution changed from colorless to yellow [21].

#### 2.2.4 Preparation of nanocarriers system

The nanocarriers were prepared using a thin-film hydration method to synthesize niosomes [22]. First, 6 % w/v of each surfactant mixture tween 80 & span 80 was mixed in a round-bottomed flask. Then, 0.01 % w/v of cholesterol was added to dissolve the mixture, and chloroform was added. The chloroform was removed by the rotary evaporator at 150 *rcf* under reduced pressure at 60 °C, resulting in the formation of a thin lipid layer. For the synthesis of NISM@BSA, 0.02 % w/v of BSA was dissolved in ultrapure water with constant stirring (150 *rcf*) for 15 min at room temperature. In two other formulations, ODNs were added to the BSA solution and stirred (150 *rcf*) for 75 min, after which the solution was added to the lipid layer slowly drop by drop (NISM@BSA-SCR, NISM@BSA-DEC). To synthesize NISM@BSA-Zn, a solution of ZnNPs (20 % v/v) was added to the BSA solution, and the resulting solution was slowly added drop by drop into the thin lipid layer. The mixture was then vigorously sonicated for 30 s with a sonicate (repeated three separate times, each time lasting 30 s). A similar procedure was followed for the NISM@BSA-SCR-Zn and NISM@BSA-DEC-Zn groups, with the addition of ZnNPs solution followed by the addition of ODNs.

#### 2.2.5 Physicochemical characterization of NPs

The physicochemical properties of the nanocarriers were characterized using DLS, FT-IR, and FESEM techniques. FT-IR spectroscopy (Bruker, Tensor 27, Biotage, Germany) was used to determine the chemical structure of BSA, NISM, NISM@B, ZnNPs, and NISM@BSA-Zn. To prepare potassium bromide (KBr) disks, 10 % of the sample and 90 % KBr were mixed and mechanically ground, and then passed in the plate form (pressure, 10 Ton) [23]. DLS was used to determine the polydispersity index (PDI), average hydrodynamic size of nanoparticles, and *zeta* potential using the Nano-Zeta sizer apparatus (Malvern Instruments, Worcestershire, UK, model Nano ZS). For each sample, 0.5 mL was diluted with ultrapure water and decanted into a Malvern sample vial before being analyzed for PDI, hydrodynamic diameter, and *zeta* potential. FESEM was used to determine the morphology and size of the nanoparticles. Gold was used to coat the samples, and FESEM (MIRA TESCAN, Czech Republic) was operated at an acceleration voltage of 15 kV and a scale of 35 KX magnification. The decoy ODNs entrapment efficiency (EE%) of the nanocarriers was calculated by diluting the nanocarriers in phosphate buffer and centrifuging them (14000 rpm, 30 min) to collect free ODNs (repeated three times). The amount of free ODNs decoy was quantified using Nanodrop at a wavelength of 260 nm [24]. The ODN entrapment efficiency (% EE) was calculated using the following formula:

$$\%\;\text{Entrapment}\;\text{Efficiency}\;\left(\%\text{EE}\right)=\frac{\text{Total}\;\text{decoy}\;\text{ODNs}-\text{Free}\;\text{decoy}\;\text{ODNs}}{\text{Total}\;\text{decoy}\;\text{ODNs}}\times 100$$

#### 2.2.6 ODNs release study

To assess the release behavior of ODNs from the nanocarrier systems (NISM@BSA-SCR, NISM@BSA-SCR-Zn, NISM@BSA-DEC, NISM@BSA-DEC-Zn), purified NISM@BSA-DEC or NISM@BSA-SCR in 1.5 mL of PBS was dispersed at pH 7.4 and 5.8, and placed in an incubator shaker maintained at 120 rpm and 37 °C. At predefined time intervals, the release medium was withdrawn and replaced with an equal amount of fresh release medium. The amount of released decoy ODN was quantified using spectrophotometry by a Nanodrop (Wilmington, USA) [18].

#### 2.2.7 ODNs release kinetic estimation

The in vitro release of ODNs was analyzed by fitting the release data to various kinetic models to assess the release mechanism and kinetics. The kinetic model was chosen based on the lowest values of the mean squared error (MSE) and Akaike's Information Criterion (AIC) [23].

#### 2.2.8 Hemocompatibility assay

Healthy human blood was obtained and 200 μg/mL EDTA was added in tubes, which were then centrifuged at 3500 rpm for 5 min at 4 °C. The plasma was separated. The RBCs were washed three times with cold PBS. The purified RBCs were then resuspended in an isotonic solution of PBS diluted to 1:10. To perform the assay, different concentrations of nanocarriers (6.25, 12, 25, 50, 100, 200 μg/mL), 2 % Triton X-100 (as a positive control showing 100 % hemolysis), and 500 μL of PBS as the negative control (0 % hemolysis) were used. 200 μL of test samples were added to tubes containing 200 μL of RBC pellets and placed in a shaker incubator at 37 °C for 4 h. After incubation, the samples were centrifuged at 4000 rpm for 10 min. The absorbance was measured at 540 nm using a spectrophotometer [18].

$$Hemolysis\;\left(\%\right)=\frac{{A\;}_{treated\;sample}-\;{A\;}_{negative\;control}}{A_{\;positive\;control}-\;A_{\;negative\;control}}\times 100$$

In this equation, *A*_*treated sample*_, *A*_*negative-control*,_ and *A*_*positive control*_ are representative of the mean absorbance of the sample, negative control, and positive control, respectively.

#### 2.2.9 Cellular uptake of nanocarrier

To determine the uptake efficiency of the nanocarrier system in NTERA-2 cells, decoy ODNs labeled with Cy3 were used. The cells were cultured in 24-well plates and incubated until reached approximately 70 % confluence. The medium was then discarded. The cells were washed twice with PBS to remove any cell debris. The NTERA-2 cells were treated with DMEM medium containing various concentrations (0.25, 0.5, 0.75, 1 μg/mL) of NISM@BSA-ODN-Zn and NISM@BSA-Zn (1 μg/mL) and incubated under standard culture conditions for 6 h. Then, the media was discarded from the wells and replaced with 500 μL of fresh complete DMEM. Finally, the cells were harvested and analyzed using flow cytometry (BD Biosciences, San Jose, CA) to determine the cellular uptake of the nanocarrier system [18].

#### 2.2.10 Cellular toxicity assay

To determine the cytotoxicity effects of the nanocarrier systems, an MTT assay was performed. The NTERA-2 cells were cultured in 96-well plates at a density of 10^4^ cells per well and incubated with a complete growth medium for 24 h. The cells were then treated with the test samples (NISM@BSA, NISM@BSA-SCR, NISM@BSA-DEC, ZnNPs, NISM@BSA-Zn, NISM@BSA-SCR-Zn, NISM@BSA-DEC-Zn) at different concentrations (0.5, 1, 3 μg/mL). In a parallel procedure to investigate the effect of radiation exposure, 5 h after treatment with the test samples, the treatment medium was removed, and 100 μL of fresh media was added to each well. Then, the plate was irradiated with a fractionated X-ray with a dose of 2 Gy. After 24 h, 20 μL of MTT solution (5 mg/mL) was added to each well, and after 4-h incubation, DMSO (100 μL) was added to each well. The absorbance at 570 nm wavelength was measured. All assays were performed in triplicate [25].

#### 2.2.11 Cell cycle assay

To assess the cell cycle, flow cytometry was performed 24 h after treatment. NTERA-2 cells were seeded in 12-well plates at a density of 3 × 10^4^ cells per well and incubated at 37 °C in a 5 % CO_2_ incubator for one day before treatment. The cells were then treated with 1 μg/mL of nanocarriers (NISM@BSA-Zn, NISM@BSA-SCR-Zn, and NISM@BSA-DEC-Zn). In a parallel procedure, 5 h after treatment, the medium in each well was removed, and 500 μL of fresh medium was added to each well. Then, the cells were immediately irradiated with fractionated X-ray with a dose of 2 Gy. After 24 h, the cells were detached using 0.05 % trypsin/EDTA and pelleted by centrifugation at 1200 rpm for 3 min. The obtained cells were washed with 50 μL of PBS and fixed with 70 % cold ethanol, followed by centrifugation to remove excess ethanol. Next, the cells were processed with 1 mL of PI Master Mix solution (40 μL PI Sigma-Aldrich, 10 μL RNase, 950 μL PBS) and incubated for 30 min at room temperature. The data gathered from flow cytometry was analyzed using *FlowJo v.7* software (Tree Star, Ashland, OR) to calculate the percentage of cells in each phase of the cell cycle [26].

#### 2.2.12 Apoptosis assay

To assess the apoptosis rate, NTERA-2 cells were seeded in 12-well plates at a density of 3 × 10^4^ cells per well and incubated for 24 h at 37 °C in a 5 % CO_2_ incubator. The cell groups were treated with different nanocarriers (NISM@BSA-Zn, NISM@BSA-SCR-Zn, and NISM@BSA-DEC-Zn) at a concentration of 1 μg/mL for 24 h. In a parallel procedure to investigate the effect of radiation, 5 h' post-treatment, the medium in each well was removed, and a fresh medium was added to each well. Then, the cells were immediately irradiated with fractionated X-ray with a dose of 2 Gy. After 24 h, the cells were washed with PBS and suspended in 100 μL of Annexin V binding buffer, and then stained with Annexin V-FITC and PI (Sigma-Aldrich). Data analysis was performed using a flow cytometry apparatus (BD Biosciences, San Jose, CA, USA) and *FlowJo* software (Tree Star, Ashland, OR) [26].

#### 2.2.13 Wound-healing (scratch) assay

To determine the migration inhibition rate, a wound-healing or scratch assay was performed. NTERA-2 cells were cultured in 24-well plates at a density of 2 × 10^4^ cells per well and incubated under standard conditions for 24 h until reached a confluence of more than 70 % of cells. Artificial wounds were created using the end of a 10 μL pipette tip (time 0 h), and detached cells and media were removed. The cells were then treated with a concentration of 0.5 μg/mL of nanocarriers (NISM@BSA-Zn, NISM@BSA-SCR-Zn, and NISM@BSA-DEC-Zn). In a parallel procedure, cells were subjected to radiation exposure by fractionated X-ray with a total dose of 2 Gy. For all plates, cell migration was monitored, and photos were taken at times 0 and 72 h. The assays were performed in triplicate. The cell migration inhibition rate was quantified using the ImageJ program [26].

### 2.3 Statistical analysis

The analysis of acquired statistical data for this research project was performed using GraphPad Prism 8 software. The results are presented as mean ± standard deviation (SD). One-way analysis of variance (ANOVA) was used for statistical analysis. Values with * representing *p* < 0.05, **p < 0.01, ***p < 0.001, and ****p < 0.0001 were considered statistically significant. All tests were performed a minimum of three times.

## 3 Results

### 3.1 Physicochemical characterization of nanocarrier system

#### 3.1.1 FT-IR spectroscopy

The FT-IR spectra of BSA, NISM, and various nanocarrier formulations (NISM@BSA, NISM@BSA-SCR, NISM@BSA-DEC, NISM@BSA-Zn, NISM@BSA-SCR-Zn, NISM@BSA-DEC-Zn) were compared, confirming the successful synthesis of the nanocarrier system (as depicted in Fig. 1, Fig. 2C). The FT-IR spectrum of BSA displayed characteristic peaks related to (amide I) bonds between 1600 and 1700 cm^−1^ and (amide II) bonds between 1500 and 1550 cm^−1^, which corresponded to the stretching vibrations of the C–O bond and bending of the N–H bond, respectively [27,28]. In the spectrum of NISM@BSA, the intensity of C–O and N–H bonds was decreased, while the N–H bending vibration in its spectrum indicated the successful synthesis of the NISM@BSA nanocarrier. The FT-IR spectra of DEC and SCR showed characteristic bands at 3440 cm^−1^ and 1650 cm^−1^, respectively, which corresponded to the stretching of N–H and C–O bonds. The spectra of NISM@BSA-DEC and NISM@BSA-SCR displayed peaks from NISM@BSA as well as overlapping peaks from DEC and SCR. The intensities in the area between 1600 and 1700 cm^−1^ were decreased in the spectra of NISM@BSA-DEC and NISM@BSA-SCR due to the incorporation of ODNs into NISM@BSA.

**Fig. 1:**
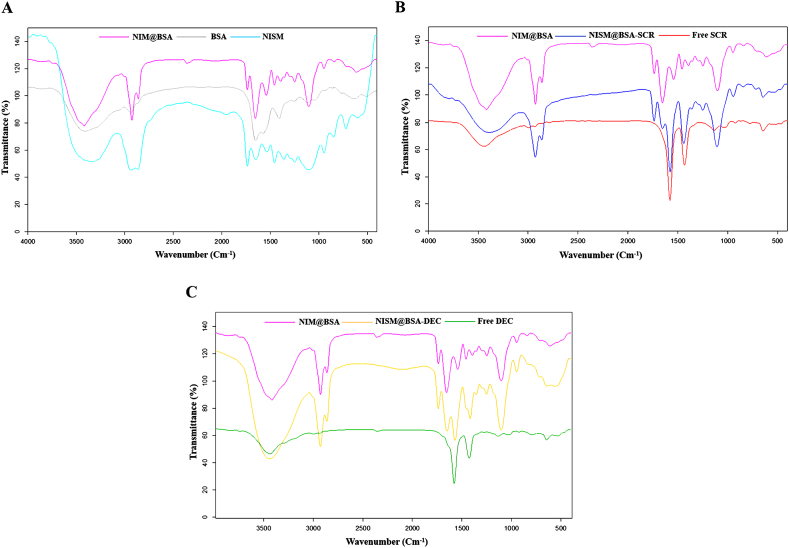
Fourier-transform infrared (FT-IR) absorption spectra for all formulations and components. (A) Comparison of FT-IR absorption peaks for NISM@BSA, BSA, and NISM. (B) Comparison of FT-IR absorption peaks for NISM@BSA, NISM@BSA-SCR, and free SCR. (C) Comparison of FT-IR absorption peaks for NISM@BSA, NISM@BSA-DEC, and free DEC.

**Fig. 2:**
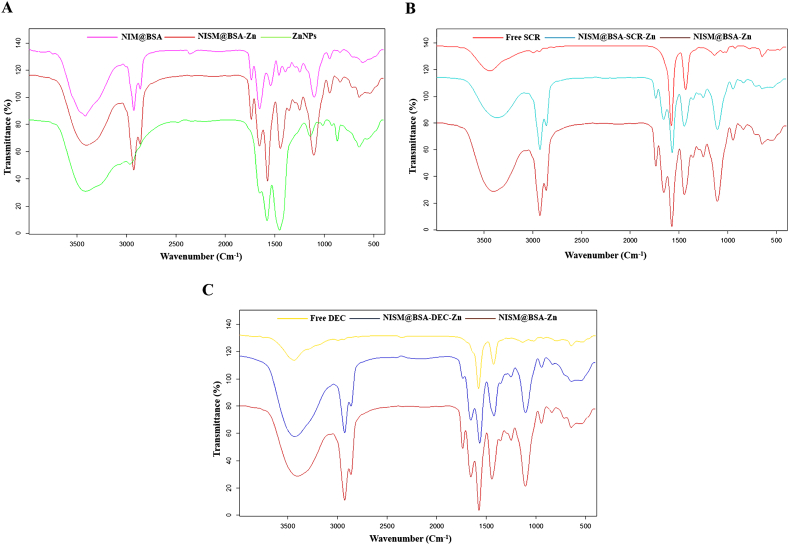
Fourier-transform infrared (FT-IR) absorption spectra for all formulations and components. (A) Comparison of FT-IR absorption peaks for NISM@BSA, NISM@BSA-Zn, and ZnNPs. (B) Comparison of FT-IR absorption peaks for NISM@BSA-Zn, NISM@BSA-SCR-Zn, and free SCR. (C) Comparison of FT-IR absorption peaks for NISM@BSA-Zn, NISM@BSA-DEC-Zn, and free DEC.

#### 3.1.2 Morphology, average hydrodynamic diameter & zeta potential

**Fig. 3:**
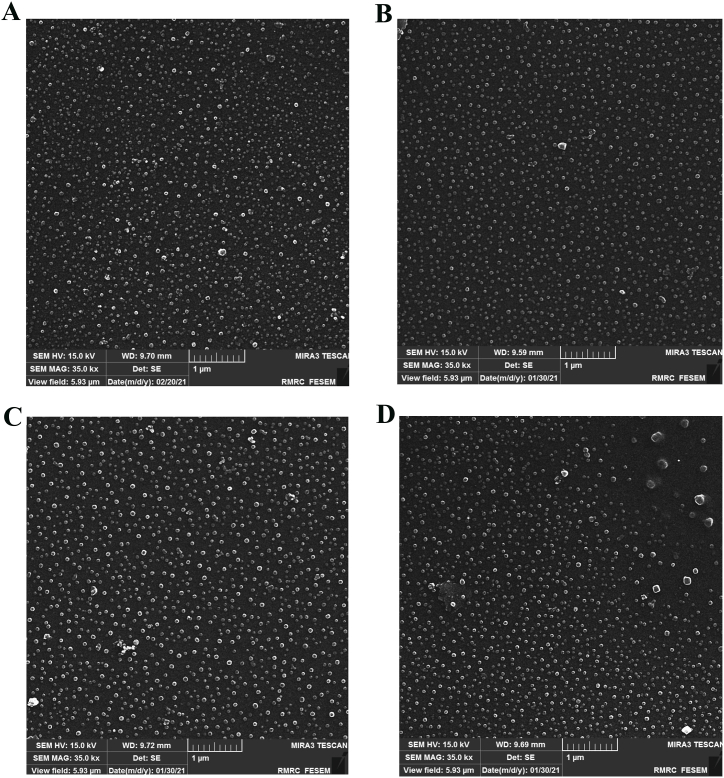
FESEM image of the (A) NISM@BSA (B) ZnNPs (C) NISM@BSA-Zn (D) NISM@BSA-ODN-Zn nanocarriers.

The morphologies of NISM@BSA, ZnNPs, NISM@BSA-Zn, and NISM@BSA-ODN-Zn were investigated using field-emission scanning electron microscopy (FESEM). As shown in Fig. 3A–D, all the nanocarriers exhibited spherical and uniform shapes. Additionally, FESEM analysis revealed that the size of NISM@BSA-ODN-Zn was larger than that of NISM@BSA. This observation indicates that the incorporation of ODN and Zn into NISM@BSA increased the size of the nanocarrier. This finding provides valuable insights into the structural characteristics of the nanocarriers, which are crucial for their optimal performance in drug delivery applications.

**Fig. 4:**
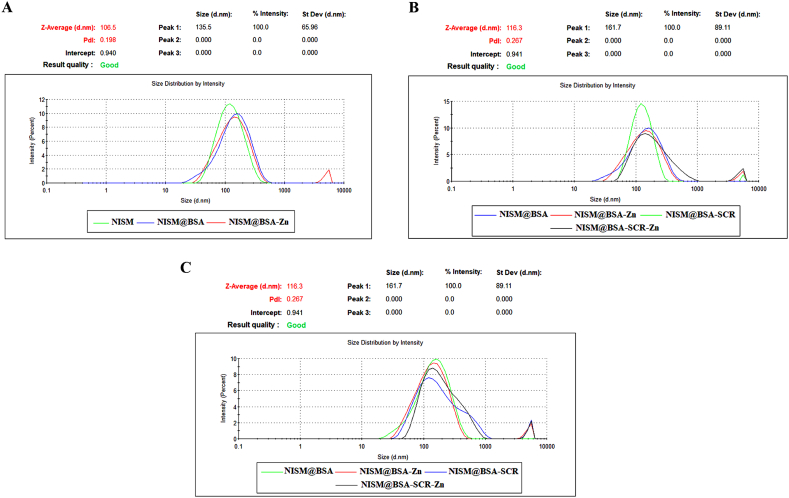
Hydrodynamic average size of NISM, NISM@BSA, NISM@BSA-SCR, NISM@BSA-DEC, NISM@BSA-Zn, NISM@BSA-SCR-Zn and NISM@BSA-DEC-Zn nanocarriers.

**Fig. 5:**
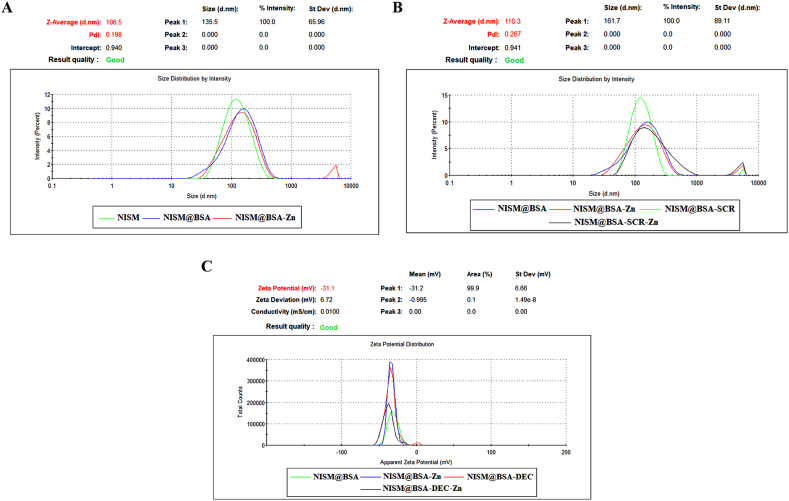
*Zeta* potential of NISM, NISM@BSA, NISM@BSA-SCR, NISM@BSA-DEC, NISM@BSA-Zn, NISM@BSA-SCR-Zn and NISM@BSA-DEC-Zn nanocarriers.

The average hydrodynamic size of nanocarriers was NISM:107.20 ± 1.21 nm, NISM@BSA:117.67±; 1.21 nm, NISM@BSA-Zn:130.43±; 1.97, NISM@BSA-SCR:143.06±; 2.62, NISM@BSA- SCR-Zn:175.47 ± 2.91 nm, NISM@BSA-DEC:158.63±; 1.52 nm, NISM@BSA- DEC-Zn: 176.67 ± 4.68 nm. *Zeta* potential results from DLS were NISM: 29.03 ± 0.51 mV, NISM@BSA: 30.73 ± 0.35 mV, NISM@BSA-Zn: 32.07 ± 1.39 mV, NISM@BSA-SCR: 34.50 ± 0.62 mV, NISM@BSA-SCR-Zn: 37.37 ± 0.45 mV, NISM@BSA-DEC: 34.97 ± 1.81 mV, NISM@BSA-DEC-Zn: 36.60 ± 0.95 mV. The differences in the size and *zeta* potential among nanocarriers were statistically significant. At last, the polydispersity index (PDI) for nanocarriers were: NISM:0.210 ± 0.012, NISM@BSA:0.261±; 0.009, NISM@BSA-Zn:0.348±; 0.010, NISM@BSA SCR:0.359 ± 0.025, NISM@BSA-SCR-Zn: 0.418 ± 0.041, NISM@BSA-DEC: 0.537 ± 0.007, NISM@BSA-DEC-Zn: 0.424 ± 0.021. The average hydrodynamic size, *zeta*-potential, and PDI of nanocarrier systems are shown in Fig. 4, Fig. 5C.

#### 3.1.3 Entrapment efficiency, behavior ODNs release, and kinetics

**Fig. 6:**
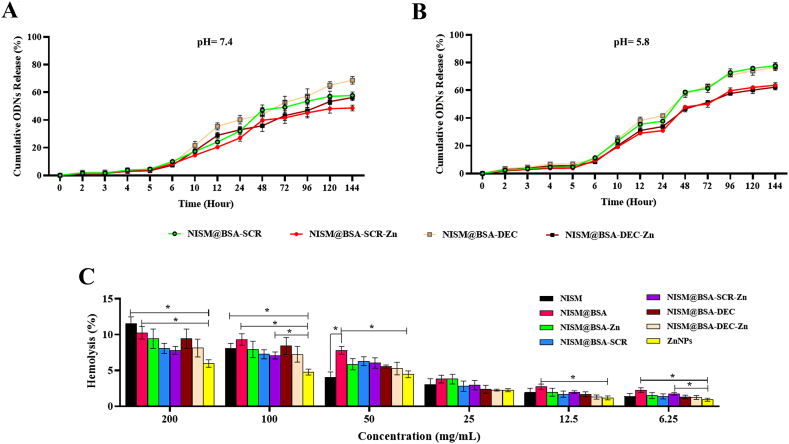
*In vitro* accumulative ODN release profile of the nanocarriers at (A) pH = 7.4 and (B) pH = 5.8. (C) Hemocompatibility assay of synthesized nanocarriers (* represents a significant difference with a difference of *p*˂0.05).

The EE% values of NISM@BSA-DEC-Zn and NISM@BSA-SCR-Zn formulations were 78.40 % + 1.22 % and 76.33 % + 1.43 %, respectively. To assess the release profiles of decoy ODN, experiments were conducted at 37 °C (human body temperature) using PBS in different pH media (7.4 and 5.8), as illustrated in Fig. 6A and B. At both pH values, the release of ODN was slow, with only 3.43%–6.89 % released within 6 h. However, sustained and controlled release behavior was observed between 8 and 120 h, with a plateau phase reached after 144 h (Table 1). The release of ODN was pH-dependent, with a faster release rate observed at pH 5.8 (0.576–0.462 %/h) than at pH 7.4 (0.420–0.436 %/h). The empirical release data was fitted to different mathematical kinetic models, and the Weibull model showed the best fit with the experimental data for NISM@BSA-SCR at both pH values (7.4 and 5.8), as well as for NISM@BSA-DEC-Zn and NISM@BSA-SCR-Zn at pH 7.4 media (Table 2). Similarly, the Gompertz model was the best fit for NISM@BSA-DEC at both pH values (7.4 and 5.8), as well as for NISM@BSA-SCR-Zn and NISM@BSA-DEC-Zn at pH 5.8 media (Table 2).

**Table 1:** Release profile parameters of the formulations.

Formulations	NISM@BSA-SCR		NISM@BSA-SCR-Zn		NISM@BSA-DEC		NISM@BSA-DEC-Zn	
pH	5.8	7.4	5.8	7.4	5.8	7.4	5.8	7.4
**ODNs release after 2h (%)**	2.41 ± 0.33	1.65 ± 0.75	1.97 ± 0.27	1.39 ± 0.63	3.52 ± 1.36	2.16 ± 0.93	2.86 ± 1.17	1.76 ± 0.76
**Cumulative ODNs release after 24h (%)**	37.77 ± 0.51	31.97 ± 2.73	30.91 ± 0.42	20.42 ± 0.65	41.64 ± 0.34	35.33 ± 2.60	33.83 ± 0.28	28.90 ± 2.12
**Maximum ODNs depletion (%)**	77.77 ± 2.26	57.63 ± 2.47	63.63 ± 1.85	48.64 ± 2.08	76.64 ± 2.49	68.68 ± 2.76	62.27 ± 2.02	56.19 ± 2.26
**Release rate (%/h)**	0.576	0.420	0.471	0.355	0.568	0.360	0.462	0.410

**Table 2:** R^2^, Akaike's information criterion (AIC), and mean squared error (MSE) parameters from fitting release data of formulations on various models. Values in bold indicate best fits.

Models		Zero Order		First Order		Higuchi		Hixson-Crowell		Weibull		Gompertz	
pH		7.4	5.8	7.4	5.8	7.4	5.8	7.4	5.8	7.4	5.8	7.4	5.8
**Formulations**	**Parameters**												
**NISM@BSA-SCR**	R^2^	0.7304	0.7622	0.8689	0.9344	0.9384	0.9430	0.8296	0.8975	**0.9872**	**0.9822**	0.9834	0.9786
	AIC	107.39	113.37	97.30	95.33	86.73	93.37	100.69	101.58	**66.34**	**78.77**	69.24	80.56
	MSE	143.05	219.21	69.57	60.43	32.70	52.53	90.39	94.44	**6.77**	**16.45**	8.81	19.77
**NISM@BSA-SCR-Zn**	R^2^	0.7304	0.7622	0.8421	0.8966	0.9384	0.9430	0.8088	0.8609	**0.9861**	0.9719	0.9794	**0.9770**
	AIC	102.65	107.75	95.16	96.09	81.98	87.75	97.83	100.25	**62.76**	79.49	67.49	**75.91**
	MSE	101.91	146.74	59.69	63.82	23.29	35.16	72.26	85.87	**5.24**	17.31	7.77	**14.18**
**NISM@BSA-DEC**	R^2^	0.7226	0.7106	0.8604	0.9093	0.9237	0.9382	0.8228	0.8633	0.9528	0.9573	**0.9547**	**0.9752**
	AIC	110.90	115.33	101.29	99.09	92.83	93.71	104.62	104.84	87.76	90.21	**86.39**	**81.79**
	MSE	183.79	252.18	92.49	79.03	50.54	53.83	117.36	119.16	31.26	37.24	**29.98**	**21.57**
**NISM@BSA-DEC-Zn**	R^2^	0.7226	0.7106	0.8294	0.8612	0.9237	0.9382	0.7980	0.8194	**0.9680**	0.9514	0.9518	**0.9725**
	AIC	105.28	109.52	98.48	99.23	87.21	87.89	100.84	102.92	**76.73**	86.20	81.67	**77.43**
	MSE	123.03	166.48	75.67	79.87	33.83	35.53	89.57	103.92	**14.21**	27.97	21.39	**15.80**

#### 3.1.4 Hemocompatibility assessment of nanocarrier systems

The obtained hemocompatibility results, as depicted in Fig. 6C, indicated that the nanocarrier systems exhibited minimal hemolytic activity. Hemolysis percentages ranging from 1 % to 12 % were observed for concentrations ranging from 6.25 to 200 μg/mL of the nanocarriers. The low hemolytic activity indicates that the nanocarrier formulations are unlikely to cause significant damage to the red blood cells or induce adverse effects on the blood components.

### 3.2 High cellular uptake of Cy3-labeled NISM@BSA-ODN-Zn in NTERA-2 cells

**Fig. 7:**
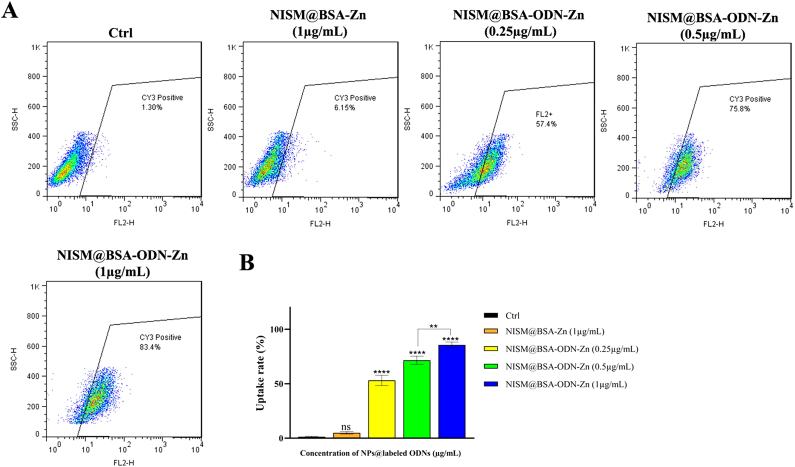
The cellular uptake rate of NISM@BSA-ODN-Zn (Cy3-labeled ODNs) into NTERA-2 cells at concentrations of 0.25, 0.5, and 1 μg/mL. NISM@BSA-Zn (1 μg/mL) as a negative control. The obtained results were analyzed by one-way ANOVA. ns (No significant), values with *p < 0.05 and ****p < 0.0001 were regarded as statistically meaningful.

The flow cytometry analysis revealed a remarkable increase in cellular uptake for Cy3-labeled NISM@BSA-ODN-Zn (85.68 %) compared to the control group (1.33 %). NTERA-2 cells efficiently took up Cy3-labeled NISM@BSA-ODN-Zn at concentrations above 0.25 μg/mL (Fig. 7A and B), with the optimal uptake observed at 0.5 μg/mL (71.56 %). The cellular uptake of Cy3-labeled NISM@BSA-ODN-Zn exhibited a dose-dependent behavior, displaying significant differences in uptake efficiency across concentrations ranging from 0.25 μg/mL to 1 μg/mL.

### 3.3 Cytotoxicity effect of nanocarriers without and under X-irradiation exposure

**Fig. 8:**
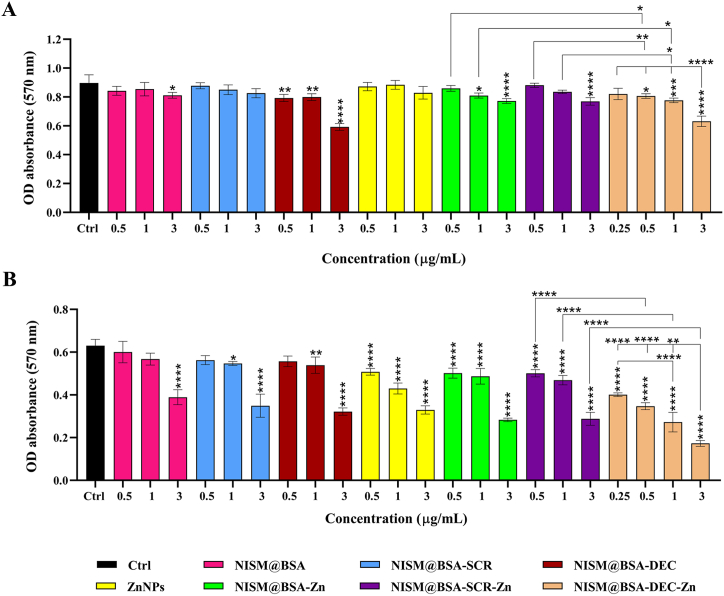
Evaluation of nanocarriers effect (NISM@ BSA, NISM@BSA-SCR, NISM@BSA-DEC, ZnNPs, NISM@BSA-Zn, NISM@BSA-SCR-Zn, NISM@BSA-DEC-Zn) on cytotoxicity. (A) without X-irradiation and (B) under X-irradiation conditions. The obtained results were analyzed by one-way ANOVA. values with *p < 0.05, **p˂0.01, ***p˂0.001, and ****p < 0.0001 were regarded as statistically meaningful.

In the absence of X-irradiation exposure, at a concentration of 0.5 μg/mL, the NISM@BSA-Zn nanocarrier showed no significant cytotoxicity effect, while at 1 μg/mL, it caused a slight increase in cytotoxicity (Fig. 8A). Similarly, 0.25 μg/mL concentrations of NISM@BSA-DEC-Zn did not significant cytotoxicity effect, but concentrations of 0.5 and 1 μg/mL led to an increase in cytotoxicity. The most significant increase in cytotoxicity was observed at concentrations of 0.5 and 1 μg/mL for NISM@BSA-DEC-Zn nanocarriers. Moreover, NISM@BSA-DEC-Zn at 3 μg/mL showed a significant difference in increasing cytotoxicity compared to concentrations of 0.25, 0.5, and 1 μg/Ml (Fig. 8A). Under X-irradiation conditions, NISM@BSA nanocarrier at concentrations of 0.5 and 1 μg/mL showed no significant difference in cytotoxicity compared to the cell control group, but there was a significant difference at the 3 μg/mL concentration. Similarly, NISM@BSA-SCR at a concentration of 0.5 μg/mL had no significant cytotoxicity effect, but the concentration of 3 μg/mL significantly increased cytotoxicity. For NISM@BSA-DEC, a concentration of 0.5 μg/mL did not show an increase in cytotoxicity, while concentrations of 1 and 3 μg/mL significantly increased cytotoxicity. In contrast, cytotoxicity was significantly increased by ZnNPs, NISM@BSA-Zn, NISM@BSA-SCR-Zn, and NISM@BSA-DEC-Zn nanocarriers at all concentrations (0.5, 1, and 3 μg/mL). Even NISM@BSA-DEC-Zn at a concentration of 0.25 μg/mL showed a significant increase in cytotoxicity (Fig. 8B). Furthermore, NISM@BSA-DEC-Zn at a concentration of 3 μg/mL revealed a significant increase in cytotoxicity compared to concentrations of 0.25, 0.5, and 1 μg/mL. Concentrations of 0.5, 1, and 3 μg/mL of NISM@BSA-DEC-Zn showed a significant increase in cytotoxicity even more than the corresponding concentrations of NISM@BSA-SCR-Zn and NISM@BSA-Zn nanocarriers (Fig. 8B). The IC50 value for the NISM@BSA-DEC-Zn, NISM@BSA-SCR-Zn, and NISM@BSA-Zn were 1.20 ± 2.33, 1.22 ± 0.25, and 2.34 ± 0.45 μg/mL, respectively. Altogether, the IC50 values for the NISM@BSA-DEC-Zn were less than other groups (Figs. S1A and B).

### 3.4 Cell cycle arrest without and under X-irradiation exposure

**Fig. 9:**
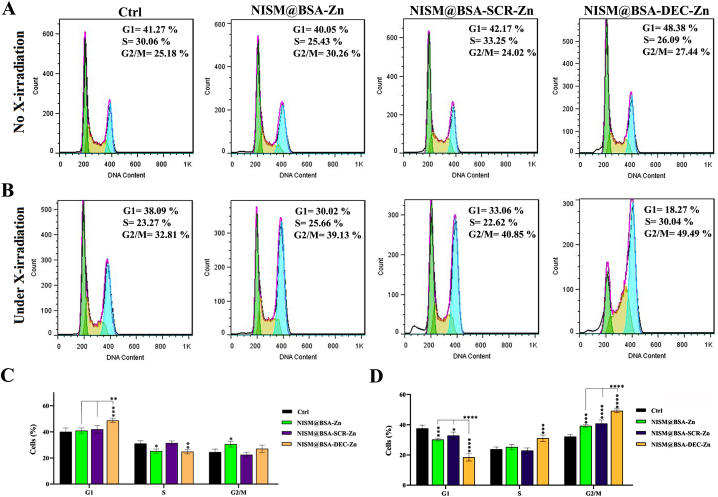
Evaluation of nanocarriers (NISM@BSA-Zn, NISM@BSA-SCR-Zn, NISM@BSA-DEC-Zn) effect on cell cycle arrest. (A) without X-irradiation and (B) under X-irradiation conditions. The obtained results were analyzed by two-way ANOVA. values with *p < 0.05, **p˂0.01, ***p˂0.001, and ****p < 0.0001 were regarded as statistically meaningful.

In the absence of X-irradiation exposure, the NISM@BSA-DEC-Zn nanocarriers induced a significantly higher percentage of cells arrested in the G1 phase compared to the other groups. Additionally, the NISM@BSA-Zn and NISM@BSA-DEC-Zn groups exhibited a lower percentage of cells in the S phase compared to the cell control group (Ctrl). Notably, the number of cells in the G2/M phase in the NISM@BSA-Zn group was slightly higher than in the Ctrl group (Fig. 9A–C). Under X-irradiation conditions (2Gy fractionation), the percentage of cells arrested in the G1 phase significantly decreased in all treatment groups compared to the Ctrl group. Among these groups, the NISM@BSA-DEC-Zn group showed the lowest percentage of cells in the G1 phase, demonstrating a more significant effect in reducing cell cycle progression at this stage compared to the groups treated with NISM@BSA-Zn and NISM@BSA-SCR-Zn nanocarriers. In contrast, the NISM@BSA-DEC-Zn group exhibited a higher percentage of cells in the S phase compared to the Ctrl group. The other treatment groups did not show significant differences in the percentage of cells in the S phase compared to the Ctrl group. Furthermore, in the G2/M phase, the percentage of cells arrested was higher in all treatment groups compared to the Ctrl group. However, the NISM@BSA-DEC-Zn group showed the most profound effect in halting cell cycle progression at this stage, as evidenced by the significantly higher number of cells arrested in G2/M compared to the groups treated with NISM@BSA-Zn and NISM@BSA-SCR-Zn nanocarriers (Fig. 9B–D).

### 3.5 Apoptosis induction without and under X-irradiation exposure

**Fig. 10:**
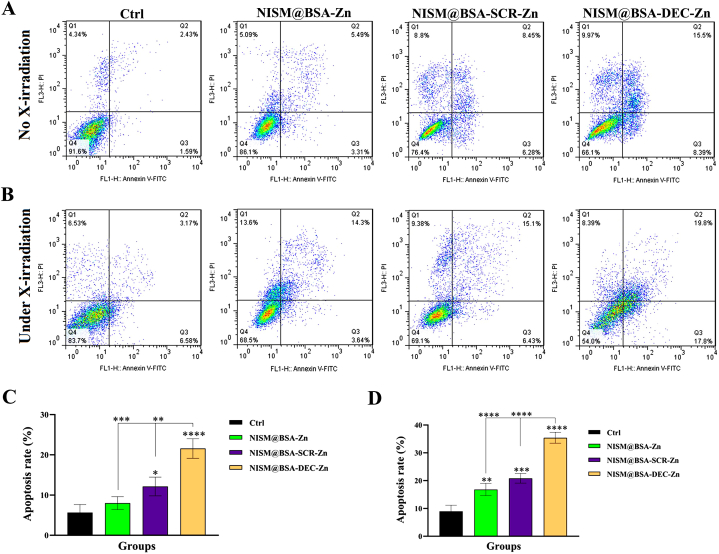
Evaluation of nanocarriers (NISM@BSA-Zn, NISM@BSA-SCR-Zn, NISM@BSA-DEC-Zn) effect on apoptosis. (A) without X-irradiation and (B) under X-irradiation conditions. The obtained results were analyzed by one-way ANOVA. values with *p < 0.05, **p˂0.01, ***p˂0.001, and ****p < 0.0001 were regarded as statistically meaningful.

In the absence of X-irradiation exposure, the cell group treated with NISM@BSA-Zn did not exhibit a significant difference in apoptosis compared to the Ctrl group. However, the group treated with NISM@BSA-SCR-Zn showed a significant increase in apoptosis compared to the Ctrl group (*p* < 0.05). Remarkably, the rate of apoptosis induced by the NISM@BSA-DEC-Zn nanocarriers revealed a highly significant difference (*p* < 0.0001) compared to the Ctrl group (Fig. 10A–C). The apoptosis rate in the NISM@BSA-DEC-Zn-treated group was also significantly different from the NISM@BSA-SCR-Zn group (*p* < 0.01) and the NISM@BSA-Zn group (*p* < 0.001). Under X-irradiation conditions, the cell group treated with NISM@BSA-Zn showed a significant increase in apoptosis compared to the Ctrl group (*p* < 0.01). Moreover, the NISM@BSA-SCR-Zn group exhibited a substantial increase in apoptosis compared to the Ctrl group (*p* < 0.001), and the NISM@BSA-DEC-Zn group demonstrated the highest apoptotic rate, significantly different from the Ctrl group (*p* < 0.0001) (Fig. 10B–D). Furthermore, the comparison of the NISM@BSA-DEC-Zn group with the NISM@BSA-SCR-Zn group revealed a highly significant difference (*p* < 0.0001), emphasizing the potent apoptotic effect of the NISM@BSA-DEC-Zn nanocarrier. Similarly, the NISM@BSA-DEC-Zn group displayed a significant difference compared to the NISM@BSA-Zn group (*p* < 0.0001).

### 3.6 Cell migration inhibition without and under X-irradiation exposure

**Fig. 11:**
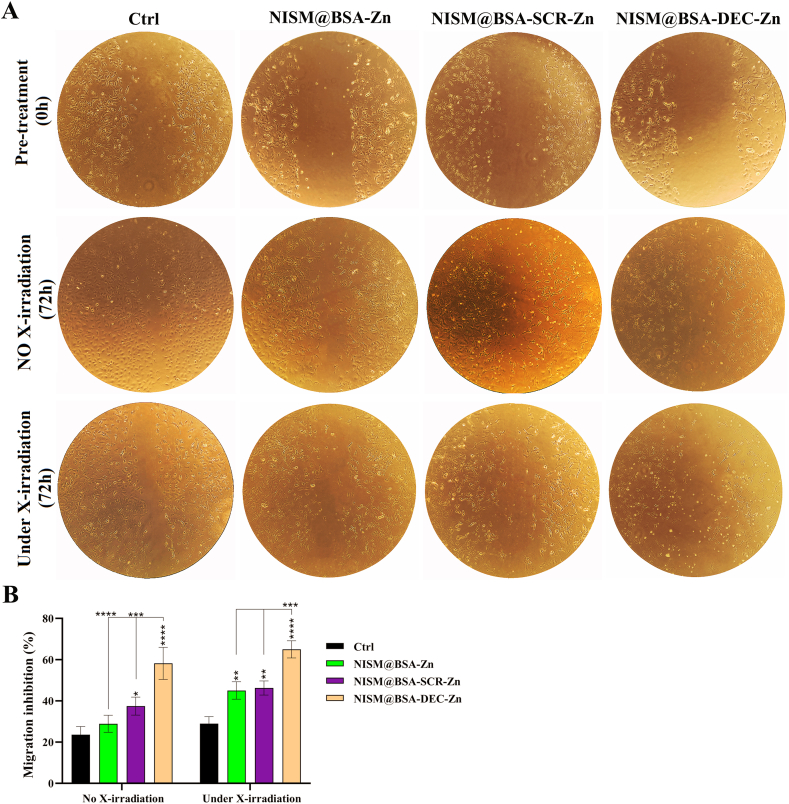
Evaluation of nanocarriers (NISM@BSA-Zn, NISM@BSA-SCR-Zn, NISM@BSA-DEC-Zn) effect on cell migration. (A) without X-irradiation and (B) under X-irradiation conditions. The obtained results were analyzed by two-way ANOVA. values with *p < 0.05, **p˂0.01, ***p˂0.001, and ****p < 0.0001 were regarded as statistically meaningful.

In the absence of X-irradiation exposure, the cell group treated with the NISM@BSA-DEC-Zn nanocarrier revealed significant cell migration inhibition (scratch repair) compared to other groups (Ctrl, NISM@BSA-Zn, and NISM@BSA-SCR-Zn). Under X-irradiation conditions, the cell group treated with the NISM@BSA-DEC-Zn nanocarrier exhibited even higher efficiency in inhibiting cell migration in comparison with the absence of X-ray conditions. The cell groups treated with both NISM@BSA-Zn and NISM@BSA-SCR-Zn nanocarriers revealed cell migration inhibition that could be due to the presence of zinc metal in the prepared nanostructure as a radiosensitizer (Fig. 11A and B).

## 4 Discussion

Cancer stem cells have been recognized as a key contributor to the metastatic spread of cancer, which has a profound impact on patient prognosis and treatment outcomes. To enhance the delivery of Sox2-Oct4 decoy ODNs, a combination of niosomes nanocarriers, BSA-coated niosomes, and zinc nanoparticles (ZnNPs) were employed to construct the hybrid nanocarriers. A thorough evaluation of the physical and chemical properties of these nanocarriers, including their dimensions, zeta potential, structure, and ODN release kinetics, was conducted. In order to ensure their suitability for potential clinical applications, the hemocompatibility of the nanocarriers was carefully assessed. Additionally, a comprehensive series of in vitro experiments were carried out to examine the effects of Sox2-Oct4 decoy ODNs enclosed within the nanocarriers on cytotoxicity, cell cycle arrest, induction of apoptosis, and cell migration inhibition in NTERA-2 cancer cells, which were utilized as a representative model of cancer stem-like cells.

The hybridization of niosomes with ZnNPs introduces distinct properties to the nanocarriers. ZnNPs within the nanocarrier have the potential to contribute to improved stability, antitumor activity, and cellular uptake, while the coating of BSA enhances biocompatibility [29]. The inclusion of ZnNPs into the niosomes also enhances the structural integrity of the nanocarriers, providing an appropriate environment for encapsulating and safeguarding the therapeutic payload, such as the Sox2-Oct4 decoy ODNs [30].

The FT-IR results confirm the successful synthesis of the nanocarrier system and the incorporation of BSA, DEC, and SCR into the NISM@BSA nanocarriers. The observed alterations in peak intensities and characteristic bands validate the formation of the desired nanocarrier structure and the successful incorporation of the components. These findings lend support to the feasibility and effectiveness of the prepared nanocarrier system for targeted delivery of the Sox2-Oct4 decoy ODNs to NTERA-2 cancer stem-like cells. The agreement of the FT-IR spectra of BSA and NISM with prior research further corroborates the dependability and replicability of the synthesis techniques employed in this study [31,32]. These findings establish a strong basis for further examinations and evaluations of the physicochemical properties, biocompatibility, and therapeutic effectiveness of the nanocarrier system against NTERA-2 cancer stem-like cells.

The results of the nanocarriers' structure, average hydrodynamic diameter, zeta potential, and polydispersity index (PDI) play a critical role in determining their suitability for drug delivery applications. These parameters offer valuable insights into the structural attributes and stability of the nanocarrier system. The spherical structure is acknowledged to enhance cellular uptake due to the augmented surface area in contact with target cells, thus potentially improving the therapeutic effectiveness of the nanocarrier system [33]. Additionally, the hydrodynamic diameter proves to be a vital parameter as it determines the stability and pharmacokinetics of the nanocarrier system. Smaller nanocarriers generally demonstrate enhanced tumor tissue penetration and extended circulation times in the bloodstream, thereby augmenting their capacity to reach the intended site and enhance therapeutic outcomes [34]. Additionally, lower PDI values indicate a narrower size distribution, which is highly desirable for drug delivery applications as it guarantees a homogeneous population of nanocarriers with consistent therapeutic efficacy. As evidenced by the findings, NISM exhibited the lowest PDI value, denoting a relatively uniform size distribution, whereas the inclusion of supplementary components resulted in marginally higher PDI values in other formulations [35]. The *zeta* potential values of the nanocarriers were also assessed via DLS and exhibited negative values across all formulations. The negative zeta potential could potentially be attributed to the presence of BSA and hydroxyl groups in cholesterol, indicating that the nanocarriers possess surface charges capable of averting aggregation and ensuring stability during storage and in physiological environments. Moreover, the integration of ZnNPs and ODNs into NISM@BSA resulted in more negative zeta potential values, indicating an augmentation in the stability and dispersion of the nanocarrier system [36]. These findings establish the foundation for further in vitro and in vivo investigations to evaluate the stability, biocompatibility, cellular uptake, and therapeutic efficacy of the nanocarrier system for potential clinical applications.

The outcomes of entrapment efficiency (EE%) of the ODNs reveal that a substantial portion of the decoy ODNs was successfully encapsulated within the nanocarriers, validating the effectiveness of the preparation technique in preserving the therapeutic payload. The release of the decoy ODNs was determined to be reliant on pH, with a swifter release rate observed at pH 5.8 (0.576%–0.462 % per hour) in contrast to pH 7.4 (0.420 %-0.436 % per hour). The pH sensitivity exhibited by the nanocarriers is of great value, as it allows them to effectively respond to the slightly acidic microenvironment present in tumors. This capability has the potential to enhance the efficiency and precision of drug release specifically within cancer cells [37]. In order to gain a deeper understanding of the release kinetics of the decoy ODNs from the nanocarriers, the experimental release data was subjected to fitting with various mathematical kinetic models. The successful fitting of the release data to these models yields valuable insights that can be utilized to optimize the design and performance of the nanocarrier system, thereby enhancing its therapeutic efficacy and potential application in cancer treatment [18]. The substantial entrapment efficiency and pH-dependent release of ODNs observed in the system demonstrate its capacity to safeguard and deliver therapeutic payloads.

When considering the potential biomedical applications of the nanocarrier systems, particularly for intravenous administration, it is imperative to thoroughly evaluate their hemocompatibility. The nanocarriers can be safely administered in the bloodstream without causing hemolysis or other detrimental effects on the blood cells due to their high level of hemocompatibility. The results of the hemocompatibility assay overall indicate that the nanocarrier systems are biocompatible with the blood components, thereby suggesting their potential as secure and promising candidates for further examination in drug delivery applications [38]. Nevertheless, to verify the safety and appropriateness of these nanocarrier systems for clinical applications, additional in vivo studies and comprehensive toxicity assessments are indispensable.

The high cellular uptake of Cy3-labeled NISM@BSA-ODN-Zn is of great significance for therapeutic applications as it allows for enhanced delivery of the encapsulated decoy ODNs to the targeted cancer stem-like cells. The selection of 0.5 μg/mL as the optimal concentration for cellular uptake (71.56 %) elucidates the dose-dependent behavior of Cy3-labeled NISM@BSA-ODN-Zn, where increasing the nanocarrier concentration resulted in a proportional increase in cellular uptake. This dose-dependent behavior is crucial in therapeutic applications as it allows for precise control over the amount of therapeutic payload delivered to the target cells, maximizing the therapeutic effect while minimizing potential toxicity to healthy cells. The notable uptake of Cy3-labeled NISM@BSA-ODN-Zn in NTERA-2 cells can be attributed to multiple factors. Primarily, the presence of a BSA-coated surface on the nanocarrier system enhances its compatibility with living organisms, thereby preventing undesired interactions with cell membranes and aiding in efficient cellular internalization. Additionally, the inclusion of ZnNPs in the nanocarriers may contribute to increased uptake by cells, owing to the role played by zinc in promoting cellular endocytosis [39].

The evaluation of cytotoxicity effects of NISM@BSA-DEC-Zn on the NTERA-2 cells revealed important insights about its potential as an anticancer agent. The obtained results demonstrated that NISM@BSA-DEC-Zn induced a concentration-dependent increase in cytotoxicity, signifying its effectiveness in inhibiting the growth and survival of NTERA-2 cells [40]. The most substantial increase in cytotoxicity was observed at concentrations of 0.5 and 1 μg/mL for NISM@BSA-DEC-Zn nanocarriers. As can be shown in the MTT results (without applying radiation), the cell viability at concentrations of 0.5, 1, and 3 μg/mL was decreased, though it did not reach IC50 in used concentrations (Figs. S1A and B). This enhanced cytotoxic effect may be attributed to the presence of the decoy ODNs encapsulated within the nanocarrier system, specifically designed to target and interfere with cancer stem-like cells' self-renewal and metastatic properties. Interestingly, the MTT test conducted under X-irradiation conditions showed varying responses to different nanocarriers. NISM@BSA and NISM@BSA-SCR at concentrations of 0.5 and 1 μg/mL did not exhibit a significant difference in cytotoxicity compared to the Ctrl group. Conversely, NISM@BSA-DEC at 0.5 μg/mL did not show a significant increase in cytotoxicity, whereas concentrations of 1 and 3 μg/mL significantly increased cytotoxicity. This highlights the potential of the decoy ODNs to exert a cytotoxic effect on NTERA-2 cells, leading to decreased cell survival. The data obtained from the study strongly suggest that NISM@BSA-DEC-Zn possesses a potent cytotoxic effect against NTERA-2 cells, potentially disrupting their growth and survival [41]. Nosrati et al. (2020) have suggested that photoabsorption and secondary electrons caused by X-irradiation can generate reactive oxygen species (ROS) that are necessary to control the fate of cancer cells during radiotherapy and chemotherapy [42]. The targeted delivery of decoy ODNs via the nanocarrier system offers a promising approach to combat cancer stem-like cells, which are known to contribute to tumor progression and resistance to conventional therapies [39].

The results obtained from cell cycle arrest indicate that the NISM@BSA-DEC-Zn nanocarriers have a potent influence on cell cycle regulation, particularly in arresting cells in the G1 and G2/M phases [13]. The enhanced G1 arrest without X-irradiation and reduced G1 arrest under X-irradiation exposure suggest that the decoy ODNs delivered by the NISM@BSA-DEC-Zn nanocarriers may interfere with key cell cycle checkpoints, leading to cell cycle arrest and potential suppression of cell growth. Additionally, the increased percentage of cells in the S phase without X-irradiation exposure suggests the activation of DNA replication, possibly indicating DNA damage repair mechanisms. The ability of NISM@BSA-DEC-Zn to modulate cell cycle progression may offer valuable insights into its potential as a targeted therapeutic strategy for cancer treatment. Previous studies revealed that cells in the late S-phase and the G2/M phase have the most potent resistance and the most sensitivity against radiation, respectively [43,44]. The cell cycle arrest observed in NTERA-2 cells treated with NISM@BSA-DEC-Zn supports its potential to disrupt cancer stem-like cells' proliferation and metastatic properties, which are essential factors in cancer progression.

The apoptosis results highlight the remarkable pro-apoptotic potential of the NISM@BSA-DEC-Zn nanocarrier in both non-irradiated and X-irradiated conditions. The higher rate of apoptosis induced by NISM@BSA-DEC-Zn can be attributed to the specific delivery of decoy ODNs targeting essential pathways involved in apoptosis regulation. This targeted approach to trigger apoptosis in cancer stem-like cells holds great promise for improving cancer therapy outcomes [11]. The findings underscore the significance of the NISM@BSA-DEC-Zn nanocarrier as a potent inducer of apoptosis in NTERA-2 cells, irrespective of X-irradiation exposure. The ability to enhance apoptosis, a crucial mechanism for eliminating cancer cells, makes NISM@BSA-DEC-Zn a promising candidate for further investigation as a targeted therapeutic agent for cancer treatment. This outcome aligns with the concept of combining therapeutic agents to enhance their overall efficacy in cancer treatment [45].

Scratch results emphasize the potent inhibitory effect of the NISM@BSA-DEC-Zn nanocarrier on cell migration, both in the absence and presence of X-irradiation exposure. The enhanced inhibition of cell migration in the NISM@BSA-DEC-Zn group can be attributed to the specific delivery of decoy oligodeoxynucleotides, which may effectively disrupt the migration and metastatic properties of cancer stem-like cells [46]. The ability of NISM@BSA-DEC-Zn to efficiently inhibit cell migration is of utmost importance in cancer therapy, as cell migration and metastasis are crucial factors contributing to cancer progression and treatment resistance. The targeted inhibition of cell migration offered by NISM@BSA-DEC-Zn holds great promise for developing novel and effective therapeutic strategies against cancer.

Altogether, the high rate of uptake of NISM@BSA-DEC-Zn by NTERA-2 cells suggests the effective efficacy of synthesized nanocarrier in drug delivery to cancer stem-like cells. This efficient delivery allows the decoy ODNs to interfere with key agents in signaling pathways within these cells, leading to antitumor effects, including cytotoxicity, cell cycle arrest, apoptosis induction, and migration inhibition. Our findings resonate with previously established research highlighting the potential of ODNs in targeting cancer stem-like cells [24,26]. Additionally, the integration of ZnNPs within nanocarriers for enhanced antitumor activity and stability finds support in existing literature [47,48]. The present study serves as a valuable stepping stone by demonstrating the promise of BSA-coated niosomes for drug delivery. While this study establishes a strong foundation in vitro, further in vivo investigations are crucial to confirm the safety and efficacy of NISM@BSA-DEC-Zn in complex biological systems. Furthermore, optimizing the nanocarrier design for specific tumor targeting and maximizing therapeutic efficacy will necessitate further research efforts.

## 5 Conclusion

In this study, niosomes coated with BSA and hybridized with zinc nanoparticles (ZnNPs) were successfully prepared and characterized. These niosomes were further loaded with Sox2-Oct4 decoy oligodeoxynucleotides (ODNs) to create the NISM@BSA-DEC-Zn nanocarrier system. In conclusion, the findings from this study open new avenues for developing advanced nanocarrier systems with potential anticancer properties against NTERA-2 cancer stem-like cells. The innovative combination of decoy ODNs, ZnNPs, and BSA-coated niosomes within the NISM@BSA-DEC-Zn nanocarrier system showcases its promising potential in the fight against cancer. However, further research, including in vivo studies and mechanistic investigations, is required to validate the safety and efficacy of this nanocarrier system in complex biological systems before its translational application in cancer treatment.

## Funding statement

The present study was supported by 10.13039/501100008323Zanjan University of Medical Sciences, Zanjan, Iran (Grant Number: A-12-1244-19 & Ethical Code: IR.ZUMS.REC.1399.412).

## Ethics approval and consent to participate

Not applicable. The NTERA-2 cell line (IBRC C10509) was purchased from the Iranian Biological Resource Center.

## Data availability statement

All supporting data included in article/supplementary material. Raw data will be made available on request.

## CRediT authorship contribution statement

**Behrooz Johari:** Writing – review & editing, Writing – original draft, Validation, Supervision, Software, Project administration, Methodology, Investigation, Funding acquisition, Formal analysis, Data curation, Conceptualization. **Shabnam Tavangar-Roosta:** Methodology, Investigation, Data curation. **Mahmoud Gharbavi:** Writing – review & editing, Software, Methodology, Investigation, Formal analysis, Conceptualization. **Ali Sharafi:** Writing – review & editing, Methodology. **Saeed Kaboli:** Methodology. **Hamed Rezaeejam:** Methodology, Investigation.

## Declaration of competing interest

The authors declare that they have no known competing financial interests or personal relationships that could have appeared to influence the work reported in this paper.

## Figures and Tables

**figs1:**
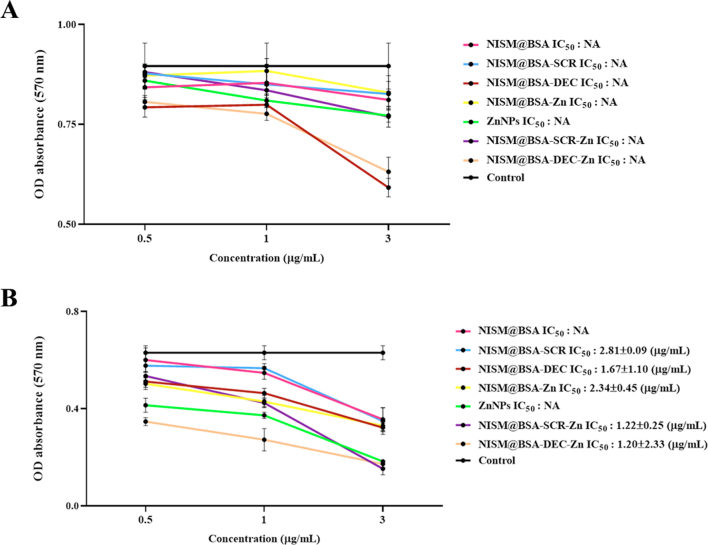


## Abbreviations

ZnNPs: Zinc nanoparticles
BSA: Bovine serum albumin
EDC: 1-(3-dimethylaminopropyl)-3-ethylcarbodiimide hydrochloride;
NHS: N-hydroxysuccinimide;
Cy3: Cyanine-3
ODNs: oligodeoxynucleotides
SCR: Scrambled
TFD: Transcription factor decoy
PS: phosphorothioate
FT-IR: Fourier transform infrared
TEM: Transmission electron microscope
SDS: Sodium dodecyl sulfate
PI: Propidium iodide

## References

[1] Siegel R.L. (2023). Cancer statistics, 2023. Ca-Cancer J. Clin..

[2] Kreso A., Dick J.E. (2014). Evolution of the cancer stem cell model. Cell Stem Cell.

[3] Lobo N.A. (2007). The biology of cancer stem cells. Annu. Rev. Cell Dev. Biol..

[4] Ali Azouaou S. (2015). Selective ROS-dependent p53-associated anticancer effects of the hypoxoside derivative rooperol on human teratocarcinomal cancer stem-like cells. Invest. N. Drugs.

[5] Schmidtova S. (2019). Disulfiram overcomes cisplatin resistance in human embryonal carcinoma cells. Cancers.

[6] Ajani J.A. (2015). Seminars in Oncology.

[7] Malecki M. (2013). TRA-1–60+, SSEA-4+, POU5F1+, SOX2+, NANOG+ clones of pluripotent stem cells in the embryonal carcinomas of the testes. J. Stem Cell Res. Ther..

[8] Pesce M., Schöler H.R. (2001). Oct-4: gatekeeper in the beginnings of mammalian development. Stem Cell..

[9] Leis O. (2012). Sox2 expression in breast tumours and activation in breast cancer stem cells. Oncogene.

[10] Barazzuol L., Coppes R.P., van Luijk P. (2020). Prevention and treatment of radiotherapy‐induced side effects. Mol. Oncol..

[11] Bigdelou Z. (2020). Role of Oct4–Sox2 complex decoy oligodeoxynucleotides strategy on reverse epithelial to mesenchymal transition (EMT) induction in HT29-ShE encompassing enriched cancer stem-like cells. Mol. Biol. Rep..

[12] Johari B. (2020). Increasing the colon cancer cells sensitivity toward radiation therapy via application of Oct4–Sox2 complex decoy oligodeoxynucleotides. Mol. Biol. Rep..

[13] Johari B., Zargan J. (2017). Simultaneous targeted inhibition of Sox2‐Oct4 transcription factors using decoy oligodeoxynucleotides to repress stemness properties in mouse embryonic stem cells. Cell Biol. Int..

[14] Moghassemi S., Hadjizadeh A. (2014). Nano-niosomes as nanoscale drug delivery systems: an illustrated review. J. Contr. Release.

[15] Puras G. (2014). A novel cationic niosome formulation for gene delivery to the retina. J. Contr. Release.

[16] Zaer M. (2023). Doxorubicin-loaded Niosomes functionalized with gelatine and alginate as pH-responsive drug delivery system: a 3D printing approach. Int. J. Biol. Macromol..

[17] Hosseini F. (2023). 3D-printing-assisted synthesis of paclitaxel-loaded niosomes functionalized by cross-linked gelatin/alginate composite: large-scale synthesis and in-vitro anti-cancer evaluation. Int. J. Biol. Macromol..

[18] Gharbavi M. (2020). NANOG decoy oligodeoxynucleotide–encapsulated niosomes nanocarriers: a promising approach to suppress the metastatic properties of U87 human glioblastoma multiforme cells. ACS Chem. Neurosci..

[19] Wellinghausen N., Kirchner H., Rink L. (1997). The immunobiology of zinc. Immunol. today.

[20] Bindu P., Thomas S. (2014). Estimation of lattice strain in ZnO nanoparticles: X-ray peak profile analysis. Journal of Theoretical and Applied Physics.

[21] Gharbavi M. (2023). Green synthesis of Zn nanoparticles and in situ hybridized with BSA nanoparticles for Baicalein targeted delivery mediated with glutamate receptors to U87‐MG cancer cell lines. Appl. Organomet. Chem..

[22] Gharbavi M. (2023). Niosomes-based drug delivery in targeting the brain tumors via nasal delivery. Nasal Drug Delivery: Formulations, Developments, Challenges, and Solutions.

[23] Jafari B. (2022). Mitigated oxidative stress and cognitive impairments in transient global ischemia using niosomal selegiline-NBP delivery. Behav. Neurol..

[24] Johari B. (2023). Combinational therapy with Myc decoy oligodeoxynucleotides encapsulated in nanocarrier and X-irradiation on breast cancer cells. Oncology Research.

[25] Johari B. (2017). Myc decoy oligodeoxynucleotide inhibits growth and modulates differentiation of mouse embryonic stem cells as a model of cancer stem cells. Anti Cancer Agents Med. Chem..

[26] Ghorbani R. (2024). Targeted anti-tumor synergistic effects of Myc decoy oligodeoxynucleotides-loaded selenium nanostructure combined with chemoradiotherapy on LNCaP prostate cancer cells. Oncology Research.

[27] Usoltsev D. (2019). Systematic FTIR spectroscopy study of the secondary structure changes in human serum albumin under various denaturation conditions. Biomolecules.

[28] Güler G. (2016). Proteolytically-induced changes of secondary structural protein conformation of bovine serum albumin monitored by Fourier transform infrared (FT-IR) and UV-circular dichroism spectroscopy. Spectrochim. Acta Mol. Biomol. Spectrosc..

[29] Wang Y. (2019). Synthesis of Zinc oxide nanoparticles from Marsdenia tenacissima inhibits the cell proliferation and induces apoptosis in laryngeal cancer cells (Hep-2). J. Photochem. Photobiol. B Biol..

[30] Ashkezari S. (2023). Antibiotic and inorganic nanoparticles co-loaded into carboxymethyl chitosan-functionalized niosome: synergistic enhanced antibacterial and anti-biofilm activities. J. Drug Deliv. Sci. Technol..

[31] Gharbavi M. (2021). Formulation and biocompatibility of microemulsion-based PMBN as an efficient system for paclitaxel delivery. Journal of Applied Biotechnology Reports.

[32] Vallieres M. (2017). Radiomics strategies for risk assessment of tumour failure in head-and-neck cancer. Sci. Rep..

[33] Xiao J., Nian S., Huang Q. (2015). Assembly of kafirin/carboxymethyl chitosan nanoparticles to enhance the cellular uptake of curcumin. Food Hydrocolloids.

[34] Gharbavi M. (2019). In vivo and in vitro biocompatibility study of novel microemulsion hybridized with bovine serum albumin as nanocarrier for drug delivery. Heliyon.

[35] Gharbavi M., Danafar H., Sharafi A. (2020). Microemulsion and bovine serum albumin nanoparticles as a novel hybrid nanocarrier system for efficient multifunctional drug delivery. J. Biomed. Mater. Res..

[36] Rashad M.M. (2021). Bovine serum albumin/chitosan-nanoparticle bio-complex; spectroscopic study and in vivo toxicological–Hypersensitivity evaluation. Spectrochim. Acta Mol. Biomol. Spectrosc..

[37] Kannaujiya V.K. (2023). Anticancer activity of NFκB decoy oligonucleotide-loaded nanoparticles against human lung cancer. J. Drug Deliv. Sci. Technol..

[38] Ali I. (2018). Hemolytic and cellular toxicology of a sulfanilamide-based nonionic surfactant: a niosomal carrier for hydrophobic drugs. Toxicology research.

[39] Ma Y. (2015). STAT3 decoy oligodeoxynucleotides-loaded solid lipid nanoparticles induce cell death and inhibit invasion in ovarian cancer cells. PLoS One.

[40] Nga N.T. (2020). Nanoliposomal cercodemasoide A and its improved activities against Ntera-2 cancer stem cells. Nat. Prod. Commun..

[41] Sadjadpour S. (2016). Antiproliferative effects of ZnO, ZnO‐MTCP, and ZnO‐CuMTCP nanoparticles with safe intensity UV and X‐ray irradiation. Biotechnol. Appl. Biochem..

[42] Nosrati H. (2020). Improved synergic therapeutic effects of chemoradiation therapy with the aid of a co-drug-loaded nano-radiosensitizer under conventional-dose X-ray irradiation. Biomater. Sci..

[43] Her S., Jaffray D.A., Allen C. (2017). Gold nanoparticles for applications in cancer radiotherapy: mechanisms and recent advancements. Adv. Drug Deliv. Rev..

[44] Liu Y. (2018). Metal-based nanoenhancers for future radiotherapy: radiosensitizing and synergistic effects on tumor cells. Theranostics.

[45] Zhao J. (2019). Enhancement of radiosensitization by silver nanoparticles functionalized with polyethylene glycol and aptamer As1411 for glioma irradiation therapy. Int. J. Nanomed..

[46] Gong N. (2018). Antisense oligonucleotide-conjugated nanostructure-targeting lncRNA MALAT1 inhibits cancer metastasis. ACS Appl. Mater. Interfaces.

[47] Elmehbad N.Y. (2023). Reinforcement of the antimicrobial activity and biofilm inhibition of novel chitosan-based hydrogels utilizing zinc oxide nanoparticles. Int. J. Biol. Macromol..

[48] Pantwalawalkar J. (2023). Stimuli-Responsive design of metal–organic frameworks for cancer theranostics: current challenges and future perspective. ACS Biomater. Sci. Eng..

